# Understanding the low uptake of long-acting reversible and permanent contraceptive methods in rural Bangladesh: A qualitative socio-ecological approach

**DOI:** 10.1371/journal.pone.0356493

**Published:** 2026-08-25

**Authors:** Sharmin Sultana Shoma, Janine Barden-O’Fallon

**Affiliations:** 1 Department of Population Sciences, University of Dhaka, Dhaka, Bangladesh; 2 Department of Maternal and Child Health, Gillings School of Global Public Health, University of North Carolina, Chapel Hill, United States of America; Addis Ababa University, ETHIOPIA

## Abstract

**Background:**

Long-acting reversible and permanent contraception methods (LARC/PM) are safe and highly efficacious; yet their uptake remains persistently low in Bangladesh. The current study sought to explore factors constraining LARC/PM uptake among married women and men of reproductive age in rural Bangladesh.

**Methods:**

This qualitative study employed purposive sampling to carry out twenty-eight in-depth interviews with married women and men, and nine key informant interviews involving family planning service providers and a religious leader in two rural communities in Cumilla district, Bangladesh. Data were analyzed thematically, guided by the socio-ecological model, to identify multilevel influences on LARC/PM uptake.

**Results:**

The findings revealed profound knowledge gaps, embodied side-effect concerns, and factual misconceptions pertaining to LARC/PM at the individual level, including fears of infertility, organ removal, and device migration, coupled with method-specific beliefs such as vasectomy-related impotence and restriction of tubal ligation to caesarean sections, largely shaped by anecdotal narratives. Interpersonal dynamics included male-dominated decision-making, spousal opposition, and pronatalist gatekeeping by female relatives. Resistance is further exacerbated by male child preference norms, stigma and myths attached to permanent methods, religious interpretations, and spousal migration at the community level. At the institutional level, provider bias, brief counselling, privacy deficits, and coercive or non-consensual practices undermined rights-based contraceptive service delivery. These were compounded by health system factors, including workforce shortages and inadequate remuneration, contributing to persistently low LARC/PM uptake.

**Conclusion:**

Low LARC/PM uptake in rural Bangladesh is shaped by a complex interplay of multilevel barriers that constrain women’s reproductive autonomy. Addressing these barriers is critical for facilitating voluntary, informed access to a full contraceptive method mix, thereby advancing reproductive health outcomes and gender equity.

## Introduction

Access to a wide range of effective contraceptive methods is essential for reducing maternal and infant mortality, enhancing reproductive health outcomes, and upholding women’s rights by enabling them to delay motherhood and space births [1]. Long-acting reversible contraceptive methods (LARC), including implants and intrauterine devices (IUDs), and permanent methods (PM), such as tubal ligation and vasectomy, offer highly effective, long-term contraceptive protection with minimal user involvement. LARC methods fulfill the need for healthier timing and spacing of pregnancies, whereas PMs are intended for those who desire no additional children [2–5]. Although LARC and PM differ in reversibility, informed consent, religious meaning, stigma, and future fertility intentions, they are analyzed together as both necessitate greater provider involvement and are embedded within overlapping structural and sociocultural constraints; this analytical grouping does not imply equivalence between a reversible and an irreversible choice. In Bangladesh, LARC/PM uptake remains disproportionately low, well below national targets, underscoring a critical gap in voluntary access to a full contraceptive method mix [3].

Bangladesh has made remarkable progress in contraceptive prevalence overall, expanding coverage from 7.7 percent in 1975–64 percent in 2022; yet it continues to grapple with a total fertility rate exceeding desired levels, a maternal mortality ratio of 115 per 100,000 live births, and an unmet need for contraception of 10% nationwide, ascending to 13–16% in certain divisions, such as Chattogram and Sylhet [3, 6]. These patterns are specifically concerning in rural areas, where unmet need (11%) exceeds urban levels (8%) and actual fertility surpasses desired fertility by nearly 0.7 children per woman, indicating that many rural women encounter barriers to fully realizing their reproductive choices [3, 7]. The contraceptive method mix remains dominated by short-acting reversible contraception methods (SARC), which require consistent adherence and timely re-supply and may be difficult to sustain in rural, resource-limited settings. Evidence from the United States suggests higher unintended pregnancy rates among SARC users compared with LARC users [5]. Expanding access to a full range of contraceptive methods, including LARC/PM, has the potential to facilitate informed, voluntary, and autonomous reproductive decision-making and contribute to enhanced maternal and child health outcomes [7–9].

Recognizing these advantages, Bangladesh prioritized scaling up LARC/PM uptake from 8.0 to 20.0% under the 4^th^ Health, Nutrition and Population Sector Program (HPNSP 2017–22). Instead, the share of LARC/PM uptake has declined from 12.1% in 1991 to 8% in 2022 [3], reflecting persistent implementation gaps including uneven service availability, reliance on the public sector, limited provider training and monitoring, logistical bottlenecks, and weak outreach in rural and underserved areas [11,12]. This, therefore, raises a critical question: what underlying factors continue to hinder LARC/PM uptake in rural Bangladesh?

Existing research in Bangladesh has concentrated primarily on overall contraceptive practices, often overlooking the nuanced dynamics in LARC/PM uptake. While most studies conducted so far focused on individual- and household-level correlates, such as age, parity, knowledge, spousal support, socioeconomic and spatial influences [10–18], less attention has been devoted to broader community, institutional, and health system influences that may affect LARC/PM decision-making. Additionally, the preeminence of quantitative analyses and the reliance on Demographic Health Survey (DHS) secondary data have constrained our understanding of why this problem persists. Although a few studies have investigated factors such as facility availability and readiness [11,12], integrated evidence capturing the interactions between demand and supply-side influences remains scarce, particularly in rural contexts. Prior studies across diverse settings imply that demand-side factors (e.g., fear of adverse effects, misconceptions, cultural beliefs) and supply-side factors (e.g., provider counselling and service access) together influence LARC/PM adoption [19–22]. These barriers, nevertheless, remain underexplored in rural Bangladesh. To date, only one known study has specifically focused on PM in Dhaka, Bangladesh [23], underscoring the need for a qualitative exploration of beliefs, contextual realities, and interacting multilevel barriers shaping LARC/PM uptake.

Against this backdrop, the present study employs a socio-ecological framework to examine influences on LARC/PM uptake in Cumilla, a district recording consistently low LARC/PM uptake within Chattogram Division [24]. By incorporating perspectives from married women, men, a religious leader, and service providers, this qualitative approach moves beyond single-level explanations to investigate the complex interplay of individual, interpersonal, community, institutional, and health-system factors shaping contraceptive decision-making in a rural, low-resource setting. The findings aim to enhance understanding of multifaceted barriers to LARC/PM access and choice and contribute to progress toward Sustainable Development Goals 3 and 5 [1].

## Methods and materials

### Theoretical orientation of the study

This study is guided by McLeroy et al.’s Socio-Ecological Model (SEM) [25], which posits a comprehensive framework for understanding how multifaceted interacting layers of intrapersonal, interpersonal, community, institutional, and health system factors influence health-seeking behavior. Rather than attributing low LARC/PM uptake entirely to individual knowledge, perceptions, or fertility intentions, SEM explicitly pays attention to the broader social and structural surroundings, including interpersonal dynamics, gendered and religious norms, socio-cultural milieu, and service accessibility that interact to facilitate or impede acceptability and access [20,22,26,27]. Applying SEM not only enables an explanation of why uptake remains low across diverse populations but also helps assess leverage points for targeted family planning initiatives to improve and sustain method uptake.

### Research strategy and design

The present study adopted a qualitative research strategy, grounded in an interpretivist epistemology, to explore how Bangladeshi rural men and women perceive and interpret the contextual factors hindering LARC/PM uptake. In order to generate contextual, experience-based insights at a single point of time, a basic interpretative qualitative design was employed using one-time, in-depth semi-structured interviews [28].

### Study area

This study was carried out in Cumilla District, which was purposively chosen due to its low uptake of LARC/PM. Based on 2022 Directorate General of Family Planning (DGFP) data, Cumilla had the lowest reported LARC/PM use (13.6%) within Chattogram Division [24], which itself accounts for one of the lowest LARC/PM uptake rates nationally (6.1%) [3]. Beyond its low uptake, this site was selected for its distinct sociocultural and programmatic context, characterized by high male out-migration that shapes household gender dynamics, as well as strong religious and community influences on reproductive decision-making. Participants were purposively recruited from two sub-districts or Upazilas of Cumilla district: Homna and Muradnagar, to capture diverse local experiences and service contexts.

### Study participants

The study population comprised three distinct groups: (1) eligible married women and men of reproductive age; (2) family planning service providers; and (3) a community religious leader (Imam). Purposive sampling was employed to recruit participants who could offer rich, contextually grounded insights into the multifaceted constraints on LARC/PM uptake. Married women and men were selected based on the eligibility criteria for LARC/PM uptake outlined in the Family Planning Manual 2017, DGFP [29], with deliberate variation in parity and fertility intentions (spacing versus limiting) to capture experiences across different reproductive life phases, including the postpartum period (Table 1). Post-menopausal women were excluded due to low fecundability and reduced need for family planning. Recruitment was facilitated by trusted local community members, such as elders and union parishad representatives, who assisted with initial village access but had no role in participant identification or eligibility screening [30]. To minimize gatekeeper bias, eligibility screening and informed consent were performed solely by the research team. Households were approached using a systematic strategy (every nth house after a random start) [31]. Of 46 eligible individuals approached, 37 consented to take part (nine refusals): twenty-two married women, six married men, eight family planning service providers (including two medical officers, three family welfare visitors, two family planning assistants, and one family planning inspector working at various tiers of the Directorate General of Family Planning), and one Imam. Refusals were more common among men, largely due to time constraints; discomfort with the sensitive topic was inferred by the research team depending on participants’ cited reasons and observed reluctance.

**Table 1:** Eligibility criteria for LARC/PM uptake based on Family Planning Manual 2017, DGFP.

Long-acting Reversible and Permanent Methods	Definition	Eligibility Criteria
**Implant**	Implant is a small flexible plastic rod which is inserted subdermally just under the skin on upper arm. It helps to prevent pregnancy for 3 years.	• Newlyweds • Women who want to delay pregnancy for a long time • For breastfeeding women: immediately after delivery
**IUD**	An IUD is a birth controlling method consisting a soft, flexible T-shaped device with a thin string on the end placed inside woman uterus, which helps prevent pregnancy for up to 10 years.	• Women who have at least one living child • Women who do not intend to have offspring for a long time or ever • Those who are breastfeeding • Those who can’t use hormonal methods.
**Vasectomy**	It is a quick and simple procedure where section of male reproductive tract is cut and sealed off in order to prevent sperm from the man’s body during ejaculation.	• Men of any age who have at least two living children • Those who never want child again.
**Female sterilization**	Female sterilization involves cutting or blocking the fallopian tubes so that sperm cannot reach the eggs.	• Women who have at least two living children. • Women having a second caesarean section. • Those who never want to have children again.

### Data collection

Data collection was done in two phases between 21 January and 20 February, 2023. The initial phase concentrated on married women and involved face-to-face in-depth interviews (IDIs) conducted to refine probing, establish rapport, and assure cultural relevance. The second phase expanded to include married men, service providers, and an imam, while continuing interviews with women, to gather data from both supply and demand sides. The first author interviewed women, providers, and an Imam, while a trained male interviewer conducted interviews with men to enhance participant comfort. Three separate semi-structured interview guides were developed for women, men, and service providers/religious leader, informed by a narrative review of published literature on barriers and facilitators to LARC/PM uptake in Bangladesh and other LMIC settings. Relevant studies were identified through PubMed, Scopus, and Google Scholar and used to inform interview domains. The guides were refined iteratively based on emerging findings, allowing both consistency and flexibility. Throughout the study, the research team remained attentive to potential power dynamics related to gender, religion, and social background and used rapport building, non-judgmental probing, confidentiality, and reflexive note-taking to reduce social desirability bias and strengthen credibility. Interviews were conducted in Bengali and lasted 23–55 minutes for women and 7–18 minutes for men. Data collection kept going until thematic saturation was reached, defined as the point at which no new themes or substantial divergence emerged. Saturation was assessed iteratively through team debriefings and was evident in the last three interviews with women and the last two with providers. Recurring patterns were apparent among male participants, although a relatively small sample may have constrained the full spectrum of viewpoints captured. The inclusion of a religious leader was intended to offer contextual insight into faith-based interpretations, rather than to represent a saturated analytic category [27].

Knowledge of LARC/PM was assessed qualitatively through open-ended questions prompting respondents to describe, in their own words, key aspects of the methods, including eligibility criteria, duration of effectiveness, mechanisms of action, advantages, potential side effects, and fertility return after removal. Additional probes assessed whether respondents could distinguish between methods and identify information sources. As the aim was to explore lived understandings, misperceptions, and contextual meanings rather than measure knowledge levels numerically, no quantitative scoring, such as knowledge scales, was used [32].

### Data analysis

Data collected through interviews were analyzed using Braun and Clarke’s thematic analysis approach [32]. All audio-recorded interviews were transcribed verbatim in Bengali. The first author translated the transcripts into English, and the second author reviewed them for accuracy, with all quotations cross-checked against the original to prevent meaning loss. Transcripts were thoroughly reviewed multiple times to develop familiarity with the information gathered and comprehend its complexity.

We adopted a hybrid coding approach, integrating inductive codes from the narratives of respondents and deductive codes derived from the socio-ecological model (SEM) and existing literature. Initial open coding was followed by focused coding to identify recurring patterns, abstract from descriptive details, and assess relevance to research objectives. These inductively derived codes and preliminary themes were subsequently interpreted and organized in relation to the levels of the SEM in order to conform to the study’s theoretical framework. Coding was conducted manually using Microsoft Excel to facilitate transparency, comparison across transcripts, and theme development. Both authors were involved in reviewing and interpreting the codes; minor adjustments and re-grouping were made through discussion until the final thematic structure was reached.

Data analysis was iterative and conducted concurrently with data collection. Preliminary analysis following the first phase of interviews informed probing in the second phase, with interpretations refined progressively until thematic saturation was reached. Broader themes and sub-themes were then developed and refined to ensure coherence and alignment with the data. Reflexive notes were maintained throughout to document the principal investigator’s positionality and analytic decisions. Relevant de-identified respondent quotations were used to support the findings, and methodological rigor was further strengthened through adherence to the Standards for Reporting Qualitative Research (SRQR) checklist [33].

### Ethical considerations

Ethical approval was obtained from the Department of Population Sciences, University of Dhaka (approval no. DPSEB/A/002/2022). Informed verbal consent was solicited from all participants following a clear explanation of the study’s purpose, the type of questions that would be asked, their voluntary and confidential nature, and the right to withdraw at any time. To safeguard anonymity and confidentiality, all transcripts and quotes presented in the findings were assigned anonymous identifiers, and gender-sensitive practices, involving separate interviews for men and women, were followed.

## Results

### Background characteristics of IDI participants

In-depth interviews with a total of 28 married individuals (twenty-two women and six men) aged 20–44 years were conducted. A summary of socio-demographic characteristics is provided in Table 2. Large families were the prevailing norm, with seventeen reporting three to five children. Among women, thirteen opted for SARC (oral pills or injectables), six adopted LARC/PM (three implants and three tubectomies), two abstained due to spousal migration, and one relied on condom uptake by her husband. Two male participants reported undergoing vasectomy.

**Table 2:** Socio-demographic characteristics of IDI participants (n = 28).

Characteristic	Women (n = 22)	Men (n = 6)	Total (n = 28)
**Age**			
20-34 years	10	0	10
≥35 years	12	6	18
**Religion**			
Muslim	21	6	27
Hindu	1	0	1
**Number of Living Children**			
1-2	11	2	13
≥3	11	4	15
**Education**			
No education to primary (≤class 5)	9	1	10
Secondary or higher	13	5	18

Note: W-Woman; M-Man: RL- Religious Leader; P- Provider.

### Factors influencing low LARC/PM uptake

Drawing on the SEM, this study delineated a range of factors shaping the LARC/PM uptake, encompassing individual, interpersonal, community, institutional, and health system domains. Table 3 provides a thematic summary of the main findings, including key themes and sub-themes to guide readers through the following details.

**Table 3:** List of themes and sub-themes presented in the results section.

Theme 1: Individual-level	Theme 2: Interpersonal-level	Theme 3: Community-level	Theme 4: Institutional-level	Theme 5: Health system-level
1.1: Lack of knowledge regarding LARC/PM 1.2: Fear of side effects and embodied experiences 1.3: LARC/PM impacts on marital relationship 1.4: Misperceptions regarding LARC/PM • LARC causes infertility • Fear of incision • LARC migrates around the body • Ligation can only be done after C-section • Vasectomy and fear of impotency	2.1: Spousal influence 2.2: Influence of family relatives and neighbors	3.1: Norms and societal stigma 3.2: Spousal migration 3.3: Male child preference norms 3.4: Cultural myths 3.5: Religious restriction and fear of punishment 3.6: Janazah (funeral) prayer is not allowed for those who uptake LARC/PM	4.1: Provider bias and gatekeeping in LARC/PM provision 4.2: Irregular visits of field workers 4.3: Family planning counselling 4.4: Provider mistreatment and violations of informed choice 4.5: Facility distance and transportation barriers	5.1: Shortages of service providers 5.2: Inadequate remuneration, incentives, and promotion opportunities for the family planning workforce

### Theme 1: Individual level factors

**Sub-theme 1.1: Lack of knowledge regarding LARC/PM.** A profound knowledge gap emerged among respondents regarding LARC/PM. Both male and female respondents demonstrated a limited understanding of what they are, who is eligible, how they work, the advantages and potential side effects, and the timeline of fertility return after removal. The majority could not differentiate between an IUD and an implant, often mistaking one for the other or assuming they were the same. Among the 22 female respondents, the implant was the most familiar (20 out of 22), while the IUD (9 out of 22) and vasectomy (8 out of 22 women) were the least known. One woman stated,

*“Look, sister, we are uneducated people. We don’t know or understand these methods. I had never heard about sterilization for either men or women before.” (W 02)*

Another woman’s narrative demonstrated a superficial familiarity with the implant, mistakenly equating its duration and mechanism with short-acting methods such as injectables (which are administered every three months):

*“I heard some inserted matchstick-type device into their hands for three months, some for six months. Menstruation, however, never stopped for those who inserted this device for three months.” (W16)*

In contrast, men’s accounts suggested even greater knowledge gaps. Among the six male participants, four were uncertain whether vasectomy applied to men. Their understanding was largely shaped by inaccurate information that was received from friends, neighbors, and relatives. A male respondent reacted with evident surprise, exclaiming,

*“Is it for men? Do men take this (vasectomy)? Even if I were given one lakh taka, I would not do this.” (M 04)*

**Sub-theme 1.2: Fear of side effects and embodied experiences.** Across interviews, fear of side effects emerged as the most frequently cited barrier among women, discouraging them from using these methods. When asked about these methods, respondents’ immediate responses typically centered on health risks, including excessive bleeding, weight gain or loss, infections, uterine complications, chronic weakness, and even serious conditions such as tumors or sexually transmitted diseases. Concerns varied by method: implants were commonly linked to abnormal bleeding leading to weakness, IUDs to malodorous discharge, infections, uterine problems, or cancer, and tubal ligation to post-surgical pain, infection, ruptured sutures, and prolonged sickness. These were not abstract fears, rather experiences grounded in women’s own bodies or those of women they were acquainted with, offering them a visceral credibility that was difficult for providers to counter. One woman explained her avoidance by drawing directly on her sister’s experience:

*“IUD is harmful – it causes foul odor, abnormal bleeding, infection. My sister used this for 8 years and told me she couldn’t bear the awful smell anymore, so she switched to pills. Again, inserting IUD inside the uterus for 10 years…could damage the uterus; that’s even more dangerous! We, women, will have no value if there’s an infection in the uterus. We will be exiled!” (W 06)*

A recurring theme was the sense of losing bodily control, particularly linked to heavier menstrual bleeding than normal, that disrupted daily life, often leading to a sense of powerlessness.

*“After inserting the implant, I was fine for 5-6 months. Then I experienced excessive bleeding. The blood flow was at its heaviest, and I screamed in pain. I couldn’t stand from weakness, and my bed would be soaked with blood every night.” (W 11)*

Notably, providers tended to attribute these concerns to client misinterpretation rather than acknowledging them as legitimate method-related experiences, reflecting a pattern of provider dismissal that may itself deepen distrust:

*“Most women who come for IUD removal complain of bleeding problems. Several blame the procedure for even minor illnesses. For example, some say they are getting thinner, others are gaining weight, or they have developed an aversion to food, all of which they attribute to the method. According to them, any health issue that arises is the fault of our method.” (P 04)*

**Sub-theme 1.3: LARC/PM impacts on marital relationship.** Concerns about the impact of LARC/PM on marital intimacy constituted a distinctive individual-level barrier, rooted in lived relational consequences rather than fear or misinformation. Nine out of twenty-two female participants raised such concerns: seven reported discomfort caused by IUD strings during intercourse, with husbands complaining of a “pinching” sensation that created tension in the relationship and often led to discontinuation, while the two described persistent post-ligation pain as a source of ongoing marital strain. One woman recounted her sister-in-law’s experience:

*“My sister-in-law took the copper T (IUD) for 3/4 months, but they faced difficulties during intimacy. Her husband disliked it, saying it caused a pinching sensation.” (W 08)*

Service providers corroborated these accounts, acknowledging that partner discomfort during intimacy and the psychological unease of having a ‘foreign object’ inside the body were frequently cited reasons for IUD avoidance.

*“Women often avoid IUD because of the string, which many husbands find uncomfortable during intimacy. As a foreign object, some who uptake sometimes feel discomfort, stating it as something ‘stuck’ inside. Many feel shy during IUD insertion. They don’t want to expose their private parts and be placed inside the sensitive area (uterus).” (P 03)*

**Sub-theme 1.4: Misperceptions regarding LARC/PM.** Misconceptions surrounding LARC/PM, often stoked by inaccurate information from peer and family networks, strongly influenced method selection. Misperceptions surrounding LARC/PM uptake were manifold below:

**LARC causes infertility.** Six female participants believed that using LARC could cause infertility. One woman attributed her sister’s subsequent infertility to LARC uptake, which strongly reinforced her own avoidance of these methods. In her words:

*“My elder sister chose a method that lasts 10 years (IUD). She suffered unbearable pain for two years and then had it removed. But she never conceived again.” (W 09)*

One service provider shared an incident that induced fear regarding this in a whole neighborhood. In his words:

*“In one case, an implant could not be located during the removal. No device was found inside her body even after the X-ray examination. The woman, who wished to have another baby, never conceived again afterwards. Can you imagine the situation? This incident fueled fear in the whole neighborhood and reinforced the perception that implants cause infertility.” (P 06)*

**Fear of incision.** Intense and persistent sensations of fear or anxiety in relation to incisions were found to have a negative impact on LARC/PM uptake. Many women expressed their reason for fear of incision, believing that body parts, particularly the kidney, might be removed during surgery for money.

*“If a part of the body is cut, something will be taken, even the kidney. Otherwise, why would they operate? Many people are afraid this will harm their health, or that something will be taken. Otherwise, why would they cut it?” (W 09)*

In response, one provider recounted an incident where a woman who had undergone ligation experienced post-surgical abdominal discomfort, which her husband attributed to organ removal.

*“It is one’s own mental thought. A few months earlier, the husband of a tubectomy user accused me of taking his wife’s kidney. Now he wants it back. His wife had a little stomach pain, for which he presumed we had taken her kidney.” (P 02)*

**LARC migrates around the body**. A few respondents expressed fear about the consequences of the device moving around their bodies. In the words of one female respondent:

*“The device (implant) does not stay in the arm; it moves around the body. My cousin had the implant inserted. She heard from her neighbor that it moves and shifts to another place. Out of fear, she removed it after three months.” (W 05)*

When asked about this statement, service providers blamed the expert’s incompetence. In the words of three service providers:

*“The implant can get embedded in the flesh of the arm. In one case, we had to get an X-ray done for a patient. The implant was there inside her arm, but it could not be grasped or removed. After that, the whole neighborhood became afraid, saying that if you take an implant, it will get stuck in the flesh like that. Actually, it had ruptured.” (P 03)*

**Ligation can only be done after C-section.** Some women perceived ligation to be available only to those who had undergone caesarean delivery, which discouraged uptake among those who delivered vaginally. Five female respondents mistakenly believed that ligation was not an option following normal delivery.

*“I didn’t go for this because all three of my children were born through normal delivery. I heard that ligation can only be done for women who have undergone caesarean delivery.” (W 09)*

Providers’ narratives supported this perception, describing that ligation was increasingly being performed during caesarean section, while standalone ligation following vaginal delivery had become less common.

*“Nowadays, Caesar ligation is more, normal ligation is reduced. Normal ligation is less common than Caesar ligation. Caesarean ligation is increasing with the number of caesarean deliveries now.” (P 06)*

**Vasectomy and fear of impotency.** Vasectomy encounters strong resistance from both men and women, driven by fears of impotence, loss of libido, generalized weakness, and social degradation. A majority of the male and female participants described vasectomy as potentially harmful to male vitality and masculinity.

*“Men lose their sexual strength and become weak. And what if, one day, we need to remarry? This is a man’s world, after all” (M 04).*

A vasectomy user directly countered these misperceptions:

*“There is no problem with doing hard work. Many are afraid of surgery because they don’t understand. They say the veins are destroyed, the body becomes weak, you will remain sick forever.” (M 03)*

A woman recounted a relative’s experience that reinforced community-level fears:

*“My relative’s husband had vasectomy abroad. Now, they sleep in separate rooms. She said, he had become impotent.” (W 18)*

Providers confirmed that fear of impotence and loss of masculinity were among the most frequently cited reasons for vasectomy avoidance:

“*Men don’t want to undergo (vasectomy), and women don’t want their husbands to opt for it. They fear they will have sexual problems, difficulties during intimacy, and that masculinity will be lost. They will become impotent. However, it has no side effects. Again, concerns that husbands work hard outside, and it will be difficult later.” (P 02)*

### Theme 2: Interpersonal level

**Sub-theme 2.1: Spousal influence.** Across the narratives of participants, a recurrent theme emerged: contraceptive decision-making, particularly around LARC/PM, was largely male-dominated. A majority of the interviewed women (15 out of 22) reported that their husbands had the final say in determining family size and approving contraception uptake, reinforced by cultural and religious norms that framed spousal permission as both marital and religious obligation.

*“Husband’s permission is required; otherwise, we will go to hell. A family planning worker once advised me to undergo ligation, but my husband forbade it. It’s a sin to do anything without his permission!” (W 06)*

Some women expressed fear of conflict, abuse, eviction, or marital breakdown as consequences of adopting contraception without spousal approval.

*“My husband will throw me out of the house! He becomes furious whenever family planning is discussed*, simply *can’t tolerate it. He said, ‘If you ever use those methods, our relationship will be over. You’ll bring ‘gazab’ (Allah’s wrath) upon our family. The unborn children will curse you for denying them life” (W 02).*

Providers similarly corroborated the deterrent effect of spousal opposition, recounting cases where husbands’ religious objections directly hindered women from accessing services:

*“Recently, a woman was scheduled to come for implant insertion. Later, she did not come. Because of my religion. I am Hindu. Her husband told her, ‘If you go to this Hindu woman for the procedure, you will become a Hindu. Our relationship will end if you do this.’ For this reason, she did not opt for implant.” (P 03)*

Simultaneously, men exercised decision-making power over contraception, while rarely taking any responsibility for it, viewing family planning as exclusively a woman’s domain:

*“Females are more likely to take these methods. They have more demand than males” (M 02).*

Providers attributed this pattern to broader patriarchal norms, stating:

*“NSV (No-Scalpel Vasectomy) uptake is least in here; men are generally unwilling to undergo this procedure. Some superstitions operate among men behind not getting NSV done. Men tend to shift the entire responsibility onto women. That’s what a patriarchal society mainly does. NSV is a simple procedure, no knife cutting, completed in the shortest possible time, and could significantly reduce the burden on women.” (P 05)*

Women’s aversion to vasectomy was also grounded in economic dependency, reflecting prevailing assumptions regarding male productivity and the household provider role:

*“I would never allow my husband to undergo vasectomy. I heard that it harms the body. If the body is not healthy, he will not be able to earn. If the head of the household is not healthy, what will happen to our family?” (W 05)*

**Sub-theme 2.2: Influence of family relatives and neighbors.** Family members, particularly mothers-in-law, were often perceived as key gatekeepers, exerting greater influence on contraceptive decisions than husbands. Several women stated that their sources of knowledge about LARC/PM were predominantly female relatives (mothers, sisters, sisters-in-law). Sharing contraceptive opinions and experiences among married women seems to be both common and socially normative, with additional input from friends and neighbors.

*“My mother-in-law, father, and mother forbade me from using* LARC/PM*. My mother said, ‘Don’t kill the child. It’s a heinous crime! You’ll become a heathen. Both you and your child will suffer consequences if you do this.” (W 07)*

Service providers also remarked that mothers-in-law are large stakeholders in the contraceptive decision-making. One provider recounted a challenging experience during a courtyard meeting, stating:

*“One day, we arranged a courtyard meeting and advised the mother-in-law to allow her daughter-in-law to adopt an implant. She refused, saying it causes dizziness. Another one raised a similar concern. Just imagine the problems this created for us! Just think what problem it created for us. At the same time, neighbors also play a role; they tend to share one’s problems with others, creating a snowball effect.” (P 01)*

### Theme 3: Community level

**Sub-theme 3.1: Norms and societal stigma.** A majority of the interviewed women stated that the oral contraceptive pills and three-month injectables were the first and most familiar methods they had ever used, which in turn propagated a cycle of continued reliance. From both female and provider participants of the study, it was found that pills and injectables were common in that community, and women are continuously taking these methods despite counselling on LARC/PM, primarily because they were the only options they were familiar with and felt confident managing.

*“Injectables and pills are the most commonly used methods among clients here. Although continual pill uptake can create many problems, many women continue to uptake, believing they can manage without issues. We offer counselling and motivation, but can’t compel anyone to uptake a method.” (P 04)*

Deviations from prevailing contraceptive norms often lead to stigmatization and discrimination, which further inhibits individuals’ demand for LARC/PM uptake. The findings of this study unfold how individuals who adopt permanent methods become targets of ridicule, often imprinted with symbolic labeling, public shaming, and social exclusion. Men who opted for vasectomy were mocked as emasculated or incapable.

*“Village people say: Oh, look what he has done! He’s gone completely. End of life. What will he even do with his wife now? She’ll leave. No other woman will marry him.” (M 02)*

Similarly, women who undergo tubal ligation are subjected to societal scorn.

*“People call me ‘pet katuni’ (woman with a cut stomach). That’s how they mock me!” (W 15, ligation user)*

**Sub-theme 3.2: Spousal migration.** Spousal migration emerged as a structural condition influencing method choice rather than merely reducing contraceptive need. Providers reported near-universal SARC use and an absence of LARC/PM use in high out-migration households, with women with migrant husbands predominantly relying on short-acting options. The prevailing reasoning, that abstinence during spousal absence dismisses the need for contraception, appears to be internalized by both women and providers, foreclosing consideration of long-acting methods. In the words of one provider:

*“Most residents here are migrants. Women whose husbands are abroad rely predominantly on short-term methods and are reluctant to switch to other options. They don’t need LARC/PM, since they remain abstinent until their husbands return.” (P 05)*

**Sub-theme 3.3: Male child preference norms.** A desire for a male child reflects a legitimate reproductive choice and should not itself be framed as a barrier to LARC/PM uptake. What the data reveal is the entrenched community-level gender norms that limit women’s reproductive autonomy from the outside, including social expectations around male inheritance, lineage continuity, and a woman’s social worth, which are not individually chosen but externally imposed, pressuring some women to continue childbearing until a male child is born. It is the structural pressure rather than individual choice that intersects with contraceptive decision-making and discourages LARC/PM uptake.

Women described the consequences of not bearing a male child in terms of status loss, marital insecurity, and social exclusion, demonstrating how deeply these norms permeate women’s sense of worth and reproductive autonomy:

“*I have two daughters. I am wishing for a son; the rest is trusting in Allah. My family is suffering a lot…regarding property distribution. We (women) are worthless without a son. I would be blessed if I had a son. Those who don’t have male child, it doesn’t make sense to use these methods.” (W 16)*

A provider’s account illustrated how these norms carry direct reproductive consequences:

*“One ligation user has three daughters. After a few months, that woman got divorced…. She has no son, that is why her husband is getting married again. But the decision was taken by both of them together." (P 07)*

**Sub-theme 3.4: Cultural myths surrounding permanent method.** Cultural myths perpetuate the belief that tubal ligation causes the death of living children as divine retribution. This conviction was so ingrained that a majority of interviewees refused ligation outright.

*“No mother wants ligation because they fear it may bring death upon their children. My husband’s sister had the surgery, and just a month later, her two-year-old son died. This is God’s punishment.” (W 10)*

Vasectomy was similarly stigmatized as causing impotence or spousal death, leading to entrenched male opposition to vasectomy uptake due to fears of emasculation and jeopardized marital prospects.

*“A neighbor’s wife died suddenly just one month after his surgery. She was healthy and fine. How could someone so healthy die so suddenly? Now, who would marry a man who can no longer have children?” (W 08)*

Religious figures often reinforce these beliefs, framing permanent methods as defiance against divine will:

*“God allows some events to happen to teach people a lesson. If He chooses to punish by taking away loved ones, no one has the strength to stop it.” (RL 01)*

One male participant, who had undergone a vasectomy and lost his wife two years following the procedure, vehemently rejected this myth:

*“Who says this nonsense? I’ll marry again this month! I don’t care about these things!!! (M 05, Vasectomy user)*

Service providers expressed frustration with clients who blame all their problems on LARC/PM:

*“Now people reproach the method for minor ailments like a cold or cough. If a child becomes sick, my child becomes sick because of ligation. When a child dies, they say ligation killed the child.” (P 04)*

**Sub-theme 3.5: Religious restriction and fear of punishment.** Religious beliefs continued to exert a strong influence on people’s attitudes and perceptions, often providing a compelling rationale against LARC/PM uptake. Our study population, Muslim-majority women and men, reported LARC/PM as heinous and impermissible, inviting divine punishment and nullifying religious devotion.

*“Do you believe in Islam? PM is directly prohibited in Islam. If I undergo the surgery, I will go to hell. Even if I pray and fast for the rest of my life, it won’t work anymore. This is disobedience to Allah!” (W 11)*

Fear and superstition compounded these religious concerns. Providers shared their clients’ religious reasoning for reluctance to PM uptake:

*“Sometimes, while we are motivating, some women stand nearby and warn, ‘Don’t adopt these methods. After death, the grave will be lifted, and the unborn children who couldn’t come into the world will punish their deceased parents. They would ask, ‘Why did you kill us? You will be punished for killing us.’” (P 04)*

The religious leader also stated his position against PM. He narrated that if they use PM to stop childbirth, the door of blessings from Allah will be closed for them, and they will be cursed by Allah. Most importantly, he mentioned the uptake of PM as “*tantamount to killing”* innocent unborn children.

*“PM users will be punished before the ‘Koborer Azab’ (punishment of the grave) because you are stopping His blessings. This also equates to murdering… a heinous crime! In the verses of the Qur’an: ‘wala taqtulo awladakum khashyata imlaqin nahnu narzuquhum wa-iyyakum inna qatlahum kana khitan kabeeran [Do not kill your children for fear of poverty, Allah will provide sustenance for them as well as for you. Verily, the killing of them is a great sin (The Qur’an 17:31).’ Allah is the owner of sustenance.” (RL 01)*

In response, PM adopters grappled with spiritual guilt, reflecting a moral struggle between doctrine and lived realities.

*“What can I say? I know it’s a sin. I just ask Allah to pardon my wrongdoing…” (W 15, Ligation user)*

A service provider pointed out challenges in engaging religious leaders in family planning promotional activities and expressed frustration with local dynamics, stating:

“*People here are religious extremists. They tend to view everything from a religious perspective. The Islamic Foundation has been advised that religious leaders and institutions should motivate and promote family planning activities, but they are not doing so.” (P 01)*

**Sub-theme 3.6: Janazah (funeral) prayer is not allowed for those who uptake LARC/PM.** Another perceived fear was that using LARC/PM would result in denial of *Janazah (funeral) prayer* and proper burial rites, as foreign objects (e.g., implant rods, surgical threads) in the body were viewed as spiritual liabilities in the afterlife. Women worried that they would be held accountable prior to God for carrying worldly items into the grave.

*“If I die with an implant inside my body, I’ll have to be accountable to Allah. That thought terrifies me. It’s forbidden to take anything worldly to the grave.” (W 06)*

Even service providers acknowledged the existence of preconceptions surrounding these methods:

*“If an IUD was inserted before death, it must be removed afterward. Otherwise, her Janazah will not be performed, nor will her body be buried.” (P 02)*

### Theme 4: Institutional level

**Sub-theme 4.1: Provider bias and gatekeeping in LARC/PM provision.** Apart from seeking information from female relatives, the study found that women trusted and valued the advice of family planning service providers. However, providers’ narratives highlighted the presence of method bias and provider-imposed eligibility restrictions in LARC/PM counselling, recommending or withholding certain methods based on their clients’ demographic characteristics and gynecologic histories. Instead of prioritizing client-centered choice, providers framed method eligibility in a number of ways that reflected restrictive presumptions. In the words of two providers:

*“To adopt LARC/PM, having at least a few children is essential. Two children, regardless of their gender, are considered sufficient. We ask our clients whether they need a gap and provide proper counselling.” (P 03)*

*“We don’t recommend IUD for women at risk of STIs or HIV, or those with uterine complications. We provide the method following a clinical examination. If any issues are identified, we don’t proceed.” (P 02)*

Concerns over the newer two-rod implant, *Femplant*, which was perceived to cause more adverse effects than earlier single-rod implants, were also voiced by some providers, along with women. These perceptions appeared to undermine trust in the method and may have further deterred adoption, particularly where counselling or procedural understanding is limited.

*“Earlier, the government provided single-rod implants, but now they supply two-rod implants like Femplant. Many women experienced side effects. In some cases, if two rods came out, people assumed that the doctor did not take them out.” (P 03)*

**Sub-theme 4.2: Irregular visits of field workers**. Seventeen out of 22 women complained that field workers visited rarely and provided minimal information regarding LARC/PM, leaving them dependent on short-acting methods. They expressed a desire to be offered wider contraceptive choices and to feel equipped with sufficient information to make an informed choice. They felt that providers should be proactive, ask the right questions, present information in an open manner, while maintaining confidentiality.

*“They visited years ago. They visit monthly for child vaccination. They never ask about family planning: which method should be used, or explain the advantages and side effects of different contraceptive methods. Women in this village are sick of taking injectables.” (W 01)*

In contrast, service providers provided a different view, claiming regular outreach and motivation efforts.

*“We visit the field regularly and motivate couples as much as possible. 20 to 25 people per day, as a part of our routine.” (P 03)*

**Sub-theme 4.3: Family planning counselling.** Family planning counselling, according to women, was consistently brief (lasting not more than 5 minutes), shallow, and poorly informative. Counselling was perceived as neither client-centered nor comprehensive, offering limited information on side effects, the full range of modern methods (especially LARC/PM), or adverse effects management, leaving women unsupported in managing repeated pregnancies.

*“Family planning people don’t explain these things properly; they don’t even come. I found her first; I was helpless with my repeated pregnancies. She didn’t explain anything, not about the implant or other available options. They don’t have that much time to tell us in detail! They never mention side effects. They always say there won’t be any problems.” (W 06)*

Additionally, privacy deficits in facility settings compounded these counselling gaps, hindering open discussion of sensitive reproductive concerns and evoking feelings of shame that discouraged women from disclosing complications or seeking clarification.

“*Again, at the hospital, there are many men and women around. I feel shy talking about my husband’s or my own problems in front of everyone. Things would be different if these conversations happened privately, in our homes. I can’t discuss the advantages and possible side effects in front of others openly.” (W 02)*

Service providers, on the other hand, perceived their counselling as adequate, claiming they allocated enough time to motivate clients, particularly new users, and offered support when problems arose.

*“We motivate our clients properly, but many don’t want to understand or accept LARC/PM. Those who have been using LARC/PM for a long time don’t require much time. But for newlyweds or those who have recently had children, we need to give them some time for motivation. We need to counsel them properly. Those who uptake LARC/PM do not need frequent visits, do not take time. If there’s a problem, I try to solve it.” (P 03)*

**Sub-theme 4.4: Provider mistreatment and violations of informed choice.** Provider mistreatment and violations of informed choices emerged as serious breaches of reproductive rights and quality of care. Women shared experiences of non-consensual method insertion, misleading counselling, and refusal to manage side effects or remove methods upon request. These infractions should be understood as failures of informed consent and accountability rather than mere barriers to future adoption. One woman shared a traumatic experience of a provider refusing to remove an implant, leading to severe health issues.

*“The blood flow was at its heaviest, and I was in constant pain- I kept screaming. My husband approached her (FWA) and explained that my condition was serious. But she insisted the implant could only be removed after 5 years, saying there was no rule to withdraw before 5 years. We are illiterate people; we didn’t understand. There won’t be a problem at all, they assured me.” (W 11)*

Another participant described that an IUD had been inserted following an abortion without her knowledge or consent:

*“They didn’t inform me at all. Following my abortion, they inserted an IUD without my knowledge or consent. I didn’t realize what had happened until I began experiencing severe abdominal pain. When I asked the doctor what had been inserted, she replied, ‘I gave you an IUD, so you won’t have more children.’ Later, I took it out by myself.” (W 03)*

A tubal ligation user reported being misled into undergoing the procedure under the false promise that it would treat her uterine prolapse, leaving her feeling devastated:

*“I underwent ligation because my uterus had dropped. I was told the procedure would help lift it. However, it didn’t lift after the surgery. Later, I was diagnosed with a tumor, and my uterus had to be removed completely. That women from family planning had promised it would fix this problem and said it would be done free of charge. That’s why I agreed. But my problem wasn’t solved. On top of that, I was diagnosed with a tumor!” (W 21)*

**Sub-theme 4.5: Facility distance and transportation barriers.** Nine out of 22 women expressed that long distances from health facilities, transportation costs, and the time needed to change multiple vehicles sometimes discourage them from seeking services.

*“No one from the government comes to talk to us. We have to go. There is no hospital nearby. A long way to go. I don’t want to go because of this. If these matters were discussed at home, I would have been more open to it.” (W 02)*

These obstacles were compounded by domestic duties, child care obligations, and restricted mobility. Irrespective of personal motivation, the concentration of LARC/PM services at Upazila facilities, rather than within communities, impedes access for rural women.

*“The center is 8 km from home…quite far. We have to change several vehicles to get there. By the time we finish household chores and send the children to school, it is already afternoon. Going at that time is not possible as it becomes dark by the time we return.” (W04)*

### Theme 5: Health-system or program level

**Sub-theme 5.1: Shortages of service providers.** Service providers highlighted substantial health workforce shortages at both the field and facility level, which compromised the delivery of family planning services, especially LARC/PM. Inadequate numbers of family welfare assistants (FWAs) and family welfare visitors (FWVs) led to overburdened staff and lowered service coverage across unions.

*“We have a substantial shortage of Family Welfare Assistants (FWAs) here, far below what is needed. Among the unions, only about half of the Family Welfare Visitors (FWVs) are in post, and some of them are currently in training.” (P 05)*

Moreover, gender-related service preferences and mismatches worsened provider availability. While the lack of male FWAs was viewed as an obstacle to engaging men in discussion about vasectomy, the absence of female surgeons deters some women from seeking services. In the words of two service providers:

*“There is a severe scarcity of manpower, even in our local office. I’m doing extra work. If there were another person, the quality of services would be enhanced. We also face a scarcity of surgeons. There is a limited number of trained surgeons available across the district. Just imagine the surgeon crisis! Another interesting fact is that many surgeons refuse to perform surgery after returning from Hajj. Moreover, there are no female surgeons in Cumilla. Many women feel uncomfortable with male touch. That’s a major barrier.” (P 01)*

*“Men in this community are more orthodox than women. And it is very sad that we don’t have any male FWAs. If male FWAs are recruited, this situation will improve.” (P 06)*

**Sub-theme 5.2: Inadequate remuneration, incentives, and promotion opportunities for the family planning workforce.** Frontline providers, particularly field-level workers, indicated low remuneration, lack of incentives, and limited promotion opportunities as major sources of provider dissatisfaction. Despite shouldering substantial responsibilities for community-based family planning outreach, they received minimal financial or professional recognition, which strongly undermined their motivation and morale.

*“Those who work at the field-level receive low pay, and their job is ‘4th class’. Yet, the entire family planning system depends on their work. They receive no additional incentives. Despite the fact that the department runs on their efforts, they have no opportunities for promotion or incentives. I believe if they were given proper incentives, it would lead to significant improvement.” (P 01)*

## Discussion

The study elucidates socio-ecological contextual factors contributing to the persistent low uptake of Long-Acting and Permanent Contraceptive Methods (LARC/PM) in rural Bangladesh. At the individual level, widespread knowledge gaps, deep-seated fears, and misconceptions appear to inhibit LARC/PM adoption.

Although the majority of participants reported having “ever heard of” LARC/PM methods, their understanding remained shallow, fragmentary, and often inaccurate, even in the presence of relatively high literacy. Knowledge was predominantly shaped by informal conversations within trusted social circles rather than provider counselling. This pattern resonates with qualitative insights from Thailand [19], where women interpret the embodied experiences of female kin and neighbors as more credible evidence of a method’s safety than formal information, pointing to a critical gap in family planning communication that providers and mass media could fill.

Fear of side effects emerged as a pervasive individual-level barrier, influencing decisions around LARC/PM even among more educated women [22,34,35]. Critically, these concerns reflect real or plausible embodied experiences, such as altered bleeding patterns, weakness, pain, infection, and IUD string discomfort, and should not be conflated with misinformation. As noted by Higgins et al. (2008) [36], contraceptive options that induce more side effects are less attractive; what this study adds is that even anticipated or vicarious experiences, circulated through community networks rather than personally encountered, carry sufficient deterrent weight. The recurring concern about IUD string discomfort during intimacy, voiced by women who had never used the method themselves, highlights how anticipated, in lieu of encountered, side effects influence couple-level acceptance [37,38], reflecting a deeper sense of bodily control that better pre-and post-insertion counselling could meaningfully address.

Distinct from these embodied experiences, participants reported a constellation of factually incorrect causal beliefs, such as LARC-induced permanent infertility, organ removal during procedures, device migration through the body, and ligation being restricted to caesarean deliveries. Return of fertility following LARC removal is typically prompt, whereas injectable (DMPA) may delay return by up to one year [39,40]; however, these distinctions were not adequately communicated in counselling, inadvertently lending credibility to infertility fears. Beliefs about covert organ removal (e.g., kidneys) for monetary gain, although clinically unfounded, appear to reflect broader mistrust in health systems and community-level rumor circulation rather than knowledge deficits alone, pointing to the need for communication strategies that address underlying distrust instead of mere repetition of informational messages.

The increased proportion of caesarean deliveries in rural Bangladesh (40.2%) [3] has expanded postpartum ligation opportunities but may have inadvertently reinforced the misconception that ligation is available only to women undergoing caesarean sections. This misperception undermines the broader potential of immediate postpartum LARC/PM provision, particularly in a context where 61% of rural births now occur in institutional settings [3]. Evidence indicates that up to 20% of postpartum women who do not initiate contraception conceive within six months of delivery, and over 10% of births occur within 24 months of a previous birth [41], emphasizing the necessity of assuring immediate postpartum LARC/PM services cover all women, regardless of delivery type or health facility.

Vasectomy remains widely underutilized, and its uptake is discouraged by misconceptions of impotence, weakness, and social degradation, in line with global as well as Bangladesh literature [23,42]. Although medically safe and highly effective permanent procedure (>99%), these deeply ingrained misconceptions continue to make vasectomy socially and culturally unacceptable to both men and women. Further study on male-specific barriers and outreach strategies for reaching men with accurate information is needed.

At the interpersonal level, patriarchal norms manifested in male dominance over contraceptive behavior. Husbands, as primary financial providers, frequently had the final say on family size and method adoption while remaining substantively disengaged from family planning practices. This led to a contradiction whereby men acknowledged women’s greater need for contraceptives, yet still framed family planning as a “woman’s domain”, leaving the burden of contraception and childbearing exclusively on women [43,44]. Spousal opposition, grounded in religious conviction, economic dependency, and fear of social consequences, further stymied women’s intention to adopt, particularly in contexts where non-compliance could lead to conflict or marital dissolution, consistent with evidence from Bangladesh and comparable settings [17,45]. These findings call out the necessity of couple-centered approaches that engage husbands in joint reproductive decision-making rather than as gatekeepers to be circumvented, with particular attention being given to the power dynamics that make such involvement structurally intricate. Female relatives, especially mothers-in-law, acted as important nodes, either reinforcing pronatalist norms [18,46] or facilitating method adoption depending on their own beliefs and exposure to FP information [47], highlighting the importance of leveraging trusted networks through peer-led information sharing programs to spread awareness.

At the community level, entrenched social norms, stigma, cultural myths, and religious strictures created formidable obstacles to the pursuit of LARC/PM services. While short-acting methods such as oral pills and injectables were viewed as socially normative, propagating a cycle of continued uptake, LARC/PM were seen as unusual or socially inappropriate. Permanent methods carried a particularly strong stigma, with users labeled as “immoral”, “impotent”, or “pet-katuni” (woman with a cut stomach), evoking shame and creating a hostile environment for method adoption. Congruent with Sharma et al. [48], men considering vasectomy feared loss of social standing and masculinity.

Spousal migration added another layer of complexity. Women, corroborated by provider accounts, described LARC/PM use as unnecessary during spousal absence. Nonetheless, this reasoning postpones rather than eliminates perceived contraceptive need, since vulnerability peaks at the moment of unpredictable spousal return when the immediate resumption of short-acting methods may not be feasible. As documented across South Asian contexts, male out-migration is associated with lower contraceptive uptake, driven by poor couple communication on family planning and reduced perceived need [49,50]. Paradoxically, the intermittent spousal contact makes LARC methods, which require no continuous adherence and no anticipation of the husband’s schedule, particularly well-suited to this context, pointing to a missed opportunity that migration-sensitive family planning programmes have yet to adequately address.

Male child preference norms operated as a community-level structural force, tying women’s social worth, marital security, and inheritance rights to their ability to bear a son, rendering LARC/PM adoption socially untenable for those without a son. A desire for a son reflects a legitimate reproductive choice; however, the constraint lies in the externally imposed norms that force her to continue having children irrespective of her own preferences or circumstances. This echoes the notion of “completion hypothesis” documented across patriarchal contexts [51–53], where sex composition of offspring takes precedence over fertility limitation, and with evidence from India and Iran linking male child preference to repeated pregnancies and stigmatization of women with no male heir [54,55]. Such findings indicate a shift from fertility limitation to fertility attainment, in which contraceptive uptake is contingent on fulfilling socially mandated reproductive obligations, suggesting that individual-level interventions alone are unlikely to be effective in the absence of efforts to address underlying gender norms.

Cultural beliefs and myths surrounding various contraceptive options paralleled evidence from broader global literature [56–58]. Participants reported fears that adopting LARC/PM would invite divine retribution, foreclose the door of blessings from God, or result in denial of *Janazah*, with PM additionally associated with fears of causing the death of a spouse or child. Religious doctrine further amplified resistance. Evidence from Bangladesh and other Muslim-majority countries [27, 59–61] indicates that although contraception is not uniformly prohibited within Islamic doctrine, localized interpretations often articulated by religious authorities, framing fertility as divinely ordained, and restricting the acceptability of PM. Contrasting evidence showing comparatively higher IUD acceptance among Muslim women in Bangladesh [18] suggests that the influence of religion is context-specific and contingent on local interpretation rather than doctrine alone. Our findings thereby imply that mosque-based messaging and religiously grounded family planning narratives may help reshape contraceptive norms.

A pronounced divergence between clients’ experiences and providers’ perspectives highlighted *institutional barriers* in the delivery of LARC/PM services. Women cited sporadic outreach, brief and inadequate counselling, lack of privacy, transportation costs, and facility distance, barriers paralleled with research from low-resources contexts [62–64] and Bangladesh notably [65,66], reflecting systematic failures in client-centered care that suppress demand irrespective of individual intent.

Conversely, providers attributed low uptake primarily to clients’ misperceptions, non-compliance, and cultural or religious beliefs while downplaying institutional shortcomings. Provider bias and limited training further restrict access to certain methods or populations, perpetuating disparities in service delivery, as documented across comparable settings [67–69]. This “blame shifting” dynamic precludes institutional actors from being accountable, shields the dysfunctional system from scrutiny, and perpetuates a deficit framing of rural women’s reproductive choices that is neither accurate nor conducive to service reformation. More critically, respondents in this study reported cases of non-consensual IUD insertion, refusal to remove implant upon request, and coercion tied to incentives or performance targets, constituting serious violations of core rights-based FP principles, including voluntary uptake, informed consent, non-coercion, and the right to discontinue [8,70]. These violations should not be dismissed as isolated instances. Coercion becomes structurally predictable when performance targets incentivize LARC/PM adoption with no sufficient safeguards for informed consent, and training alone is unlikely to address individual women’s experiences of right violations. This calls for accessible grievance mechanisms, independent oversight, and enforced provider accountability, moving rights-based family planning from rhetoric to institutional reality.

Bangladesh’s contraceptive landscape has undergone a marked transition from substantial reliance on LARC and PM in 1970s and early 1980s to constant dominance of SARCs since late 1980s [3,24]. This programmatic reorientation is partly reinforced by persistent shortages of trained workforce, excessive workloads, inadequate remuneration, weak incentive structures, and limited promotion opportunities, subtly reorienting providers toward easier, short-term methods, which is consistent with studies conducted worldwide [26,71,72]. Furthermore, Bangladesh’s health system, progressively geared toward curative services (including deliveries, and emergency management), and characterized by weak linkages with community-level FWAs [66], restricts responsiveness to clients’ family planning demands and adds to LARC/PM under-provision, suggesting the necessity to realign program priorities, strengthen workforce support, and community -facility linkages to improve the reach and sustainability of FP programs.

### Strengths and limitations

This study addresses a critical gap in understanding persistently low uptake of long-acting reversible and permanent contraceptive methods (LARC/PM) in rural Bangladesh using a socio-ecological framework and rich qualitative data from multiple stakeholders. Nevertheless, several limitations should be considered. First, the findings are based on data from Cumilla and may not fully capture variations across different regions or urban areas of Bangladesh, where sociocultural norms, healthcare access, and service availability may vary. However, the purposive selection of the study site enables in-depth contextual understanding and analytical transferability to similar rural contexts. Second, the relatively small number of male participants and shorter interview durations constrained the depth of male viewpoints and should be taken into account when analyzing gender-related insights. Third, the sample was predominantly Muslim, and the religious viewpoints were derived from a single religious leader, which may not reflect the diversity of religious interpretations across communities. Fourth, combining LARC and PM under a single analytical framework may obscure notable distinctions in reversibility, informed consent, stigma, religious meaning, and future fertility goals, meriting dedicated investigation in future studies. Finally, despite careful translation procedures, some degree of meaning loss or subtle nuance alteration may have occurred during translation from Bengali to English [73].

## Conclusion

This study demonstrates that low LARC/PM uptake in rural Bangladesh cannot be explained by individual knowledge deficits alone; rather, it is embedded in fears and misperceptions, patriarchal control over decision-making, community stigma, son preference norms, spousal migration, religious influences, and systemic shortcomings in informed consent and quality of care operating across individual, interpersonal, community, institutional, and health system levels. These findings reveal a central tension between deeply ingrained socio-cultural structures and shortcomings in health system responsiveness that limit women’s reproductive autonomy. Addressing these barriers therefore warrants a multi-pronged approach that moves beyond information campaigns. Family planning and integrated health initiatives should prioritize the meaningful involvement of men and religious leaders, promote shared decision-making, strengthen couple-centered and rights-based counselling, and enforce provider accountability to advance reproductive health and gender equity under Sustainable Development Goals 3 and 5.

## References

[1] United Nations Department of Economic and Social Affairs, Population Division. Family planning and the 2030 Agenda for Sustainable Development: Data Booklet. New York: UN DESA. 2019. https://digitallibrary.un.org/record/3832669/files/familyPlanning_DataBooklet_2019.pdf

[2] World Health Organization. Medical eligibility criteria for contraceptive use. 6th ed. Geneva: World Health Organization. 2025.

[3] National Institute of Population Research and Training (NIPORT), I C F ICF. Bangladesh Demographic and Health Survey 2022: Final Report. Dhaka, Bangladesh, and Rockville, Maryland, USA: National Institute of Population Research and Training (NIPORT) and ICF. 2024. https://dhsprogram.com/pubs/pdf/FR386/FR386.pdf

[4] MEASURE Evaluation. The future of long-acting and permanent methods of contraception in Bangladesh. Chapel Hill (NC): MEASURE Evaluation. 2014. https://www.measureevaluation.org/publications/fs-14-131.html

[5] Winner B, Peipert JF, Zhao Q, Buckel C, Madden T, Allsworth JE, et al. Effectiveness of long-acting reversible contraception. N Engl J Med. 2012;366(21):1998–2007. doi: 10.1056/NEJMoa1110855 22621627

[6] World Health Organization, UNICEF, United Nations Population Fund, World Bank Group, United Nations Department of Economic and Social Affairs, Population Division. Trends in maternal mortality 2000–2023: estimates by WHO, UNICEF, UNFPA, World Bank Group and UNDESA/Population Division. Geneva: World Health Organization. 2025. https://www.who.int/publications/i/item/9789240108462

[7] Noor FR, Rahman MM, Rob U, Bellows B. Unintended pregnancy among rural women in Bangladesh. Int Q Community Health Educ. 2011;32(2):101–13. doi: 10.2190/IQ.32.2.b 23000458

[8] Hubacher D, Spector H, Monteith C, Chen P-L, Hart C. Long-acting reversible contraceptive acceptability and unintended pregnancy among women presenting for short-acting methods: a randomized patient preference trial. Am J Obstet Gynecol. 2017;216(2):101–9. doi: 10.1016/j.ajog.2016.08.033 27662799 PMC5479328

[9] Setty-Venugopal V, Upadhyay UD. Birth spacing. Three to five saves lives. Popul Rep L. 2002;(13):1–23. 12469475

[10] Akter S, Khan MMH, Hossain AHMK, Uddin MSG, Haque MA. Prevalence and factors associated with the use of long-acting reversible and permanent contraceptive methods among women who desire no more children in Bangladesh. Contracept Reprod Med. 2025;10(1):32. doi: 10.1186/s40834-024-00331-6 40301957 PMC12039218

[11] Dana GPT, Sutopa TS, Afroz S. Disparities in readiness of health facilities to provide long-acting reversible contraceptives (LARCs) and permanent methods (PMs) in Bangladesh. Heliyon. 2023;9(4):e15349. doi: 10.1016/j.heliyon.2023.e15349 37095943 PMC10122008

[12] Khan MN, Akter S, Islam MM. Availability and readiness of healthcare facilities and their effects on long-acting modern contraceptive use in Bangladesh: analysis of linked data. BMC Health Serv Res. 2022;22(1):1180. doi: 10.1186/s12913-022-08565-3 36131314 PMC9490900

[13] Mehzabin S, Kutubi A, Elahi MM, Bar D, Manni UJA, Islam M. Factors Responsible for Premature Discontinuation of Long Acting Reversible Contraceptives A Study Conducted In Model Family Planning Clinic of Dhaka Medical College Hospital Over A Period of One Year. J Dhaka Med Coll. 2020;28(1):112–8. doi: 10.3329/jdmc.v28i1.45766

[14] Mohsena M, Tomalika N, Afroz S. Factors affecting long acting and permanent contraceptive methods (LAPM) use among women of reproductive age in Bangladesh: evidence from Bangladesh demographic health survey. Int J Curr Med Pharm Res. 2017;3(8):2268–73.

[15] Uddin MN, Ara R. Prevalence of long acting and permanent contraceptive methods among married population in a selected rural community. J Armed Forces Med Coll. 2015;9(2):93–9. doi: 10.3329/jafmc.v9i2.21846

[16] Ugaz JI, Chatterji M, Gribble JN, Banke K. Is Household Wealth Associated With Use of Long-Acting Reversible and Permanent Methods of Contraception? A Multi-Country Analysis. Glob Health Sci Pract. 2016;4(1):43–54. doi: 10.9745/GHSP-D-15-00234 27016543 PMC4807748

[17] Uddin J, Hossin MZ, Pulok MH. Couple’s concordance and discordance in household decision-making and married women’s use of modern contraceptives in Bangladesh. BMC Womens Health. 2017;17(1):107. doi: 10.1186/s12905-017-0462-3 29121901 PMC5680601

[18] Nahar KN, Fatima P, Dewan F, Yesmin A, Laila TR, Begum N, et al. Acceptability and Feasibility of Postpartum Intra Uterine Contraceptive Device Insertion in Bangabandhu Sheikh Mujib Medical University, Dhaka, Bangladesh. Bangladesh Med J. 2019;47(3):25–31. doi: 10.3329/bmj.v47i3.43495

[19] Gedeon J, Hsue SN, Walsh M, Sietstra C, MarSan H, Foster AM. Assessing the experiences of intra-uterine device users in a long-term conflict setting: a qualitative study on the Thailand-Burma border. Confl Health. 2015;9(1):6. doi: 10.1186/s13031-015-0034-9 25691916 PMC4330595

[20] Goldberg D, Sahgal B, Beeson T, Wood SF, Mead H, Abdul-Wakil A, et al. Patient perspectives on quality family planning services in underserved areas. Patient Experience Journal. 2017;4(1):54–65. doi: 10.35680/2372-0247.1194

[21] Mahato PK, Sheppard ZA, van Teijlingen E, De Souza N. Factors associated with contraceptive use in rural Nepal: Gender and decision-making. Sex Reprod Healthc. 2020;24:100507. doi: 10.1016/j.srhc.2020.100507 32200229

[22] W A, B T, M K, S G, K K. Acceptance and Positive Attitude Increased Utilization of Long Acting and Permanent Family Planning Methods Among Reproductive Age Group Women from Debre Berhan District, Ethiopia: Quantitative and Qualitative Analysis. J Community Med Health Educ. 2017;07(04). doi: 10.4172/2161-0711.1000541

[23] McEachran J. Do women support vasectomy?: attitudes to vasectomy among women attending Marie Stopes Clinic Society in Dhaka, Bangladesh. Marie Stopes International. 2002.

[24] Directorate General of Family Planning. MIS. DGFP. http://mis.dgfp.gov.bd Accessed 2022 November 27.

[25] McLeroy KR, Steckler A, Bibeau D. The social ecology of health promotion interventions. Health Educ Q. 1988;15(4):351–77. 3068205 10.1177/109019818801500401

[26] Schölmerich VLN, Kawachi I. Translating the Social-Ecological Perspective Into Multilevel Interventions for Family Planning: How Far Are We? Health Educ Behav. 2016;43(3):246–55. doi: 10.1177/1090198116629442 27105643

[27] Yillah RM, Bull F, Sawaneh A, Reindorf B, Turay H, Wurie HR, et al. Religious leaders’ nuanced views on birth spacing and contraceptives in Sierra Leone - qualitative insights. Contracept Reprod Med. 2024;9(1):40. doi: 10.1186/s40834-024-00301-y 39180098 PMC11342541

[28] Bryman A. Social research methods. 4th ed. New York, USA: Oxford university press. 2012.

[29] পরিবার পরিকল্পনা অধিদপ্তর, Hasi M. পরিবার পরিকল্পনা পদ্ধতি বিষয়ক তথ্য সহায়িকা (ফ্লিপচার্ট). http://etoolkits.dghs.gov.bd/toolkits/bangladesh-fp/fp-27 Accessed 2023 October 1.

[30] Garnett A, Northwood M. Recruitment of Community-Based Samples: Experiences and Recommendations for Optimizing Success. Can J Nurs Res. 2022;54(2):101–11. doi: 10.1177/08445621211060935 34841904 PMC9109582

[31] Levy PS, Lemeshow S. Sampling of populations: methods and applications. 4th ed. Hoboken: Wiley & Sons. 2008. doi: 10.1002/9780470374597

[32] Braun V, Clarke V. Using thematic analysis in psychology. Qual Res Psychol. 2006;3(2):77–101. doi: 10.1191/1478088706qp063oa

[33] O’Brien BC, Harris IB, Beckman TJ, Reed DA, Cook DA. Standards for reporting qualitative research: a synthesis of recommendations. Acad Med. 2014;89(9):1245–51. doi: 10.1097/ACM.0000000000000388 24979285

[34] Jogiya PD, Lodhiya KK, Chavada P. Assessment of awareness and beliefs regarding intra uterine device amongst its former users attending tertiary care centre in Gujarat. International Journal of Medical Research & Health Sciences. 2015;4(2). doi: 10.5958/2319-5886.2015.00062.4

[35] Kamal SMM, Islam MA. Contraceptive use: socioeconomic correlates and method choices in rural Bangladesh. Asia Pac J Public Health. 2010;22(4):436–50. doi: 10.1177/1010539510370780 20659903

[36] Higgins JA, Hirsch JS. Pleasure, power, and inequality: incorporating sexuality into research on contraceptive use. Am J Public Health. 2008;98(10):1803–13. doi: 10.2105/AJPH.2007.115790 18703457 PMC2636476

[37] Aoun J, Dines VA, Stovall DW, Mete M, Nelson CB, Gomez-Lobo V. Effects of age, parity, and device type on complications and discontinuation of intrauterine devices. Obstet Gynecol. 2014;123(3):585–92. doi: 10.1097/AOG.0000000000000144 24499755

[38] Wiebe ER. A comparison of male partners’ reactions to different intrauterine device strings. Int J Gynaecol Obstet. 2015;128(3):267. doi: 10.1016/j.ijgo.2014.09.020 25433392

[39] Hov GG, Skjeldestad FE, Hilstad T. Use of IUD and subsequent fertility--follow-up after participation in a randomized clinical trial. Contraception. 2007;75(2):88–92. doi: 10.1016/j.contraception.2006.09.010 17241835

[40] Doll H, Vessey M, Painter R. Return of fertility in nulliparous women after discontinuation of the intrauterine device: comparison with women discontinuing other methods of contraception. BJOG. 2001;108(3):304–14. doi: 10.1111/j.1471-0528.2001.00075.x 11281473

[41] Bairagi R, Rahman M. Contraceptive failure in Matlab, Bangladesh. Int Fam Plann Perspect. 1996;22(1):21–5. doi: 10.2307/2950798

[42] Kriel Y, Milford C, Cordero J, Suleman F, Beksinska M, Steyn P, et al. Male partner influence on family planning and contraceptive use: perspectives from community members and healthcare providers in KwaZulu-Natal, South Africa. Reprod Health. 2019;16(1):89. doi: 10.1186/s12978-019-0749-y 31238960 PMC6593556

[43] Cebeci Save D, Erbaydar T, Kalaca S, Harmanci H, Cali S, Karavus M. Resistance against contraception or medical contraceptive methods: a qualitative study on women and men in Istanbul. Eur J Contracept Reprod Health Care. 2004;9(2):94–101. doi: 10.1080/13625180410001715663 15449821

[44] Mistik S, Naçar M, Mazicioğlu M, Cetinkaya F. Married men’s opinions and involvement regarding family planning in rural areas. Contraception. 2003;67(2):133–7. doi: 10.1016/s0010-7824(02)00459-6 12586323

[45] Akamike IC, Madubueze UC, Okedo-Alex IN, Anyigor CJ, Azuogu BN, Umeokonkwo CD, et al. Perception, pattern of use, partner support and determinants of uptake of family planning methods among women in rural communities in Southeast Nigeria. Contracept Reprod Med. 2020;5:14. doi: 10.1186/s40834-020-00120-x 32884833 PMC7461266

[46] Mitchell A, Puri MC, Dahal M, Cornell A, Upadhyay UD, Diamond-Smith NG. Impact of Sumadhur intervention on fertility and family planning decision-making norms: a mixed methods study. Reprod Health. 2023;20(1):80. doi: 10.1186/s12978-023-01619-7 37231469 PMC10211300

[47] Pradhan MR, Mondal S. Examining the influence of mother-in-law on family planning use in South Asia: insights from Bangladesh, India, Nepal, and Pakistan. BMC Womens Health. 2023;23(1):418. doi: 10.1186/s12905-023-02587-7 37553598 PMC10410985

[48] Sharma S, Sharma R, Choudhary S. A study of male sterilization with no scalpel vasectomy. JK Sci. 2014;16(2):67.

[49] Mukherjee S, Mahapatra B, Saggurti N. Why women do not use contraceptives: Exploring the role of male out-migration. PLoS One. 2021;16(3):e0249177. doi: 10.1371/journal.pone.0249177 33784370 PMC8009410

[50] Samanta R, Munda J. Husband’s migration status and contraceptive behaviors of women: evidence from Middle-Ganga Plain of India. BMC Womens Health. 2023;23(1):180. doi: 10.1186/s12905-023-02325-z 37061675 PMC10105934

[51] Jiang Q, Li Y, Sanchez-Barricarte JJ. Fertility intention, son preference, and second childbirth: survey findings from Shaanxi province of China. Soc Indic Res. 2016;125(3):935–53. doi: 10.1007/s11205-015-0875-z 28769144 PMC5536174

[52] Jha P, Kesler MA, Kumar R, Ram F, Ram U, Aleksandrowicz L, et al. Trends in selective abortions of girls in India: analysis of nationally representative birth histories from 1990 to 2005 and census data from 1991 to 2011. Lancet. 2011;377(9781):1921–8. doi: 10.1016/S0140-6736(11)60649-1 21612820 PMC3166246

[53] Babalola S, John N. Factors underlying the use of long-acting and permanent family planning methods in Nigeria: a qualitative study. 2012. https://www.cabidigitallibrary.org/doi/full/10.5555/20133342876

[54] Sharma M, Joshi S, Nagar O, Sharma A. Determinants of intrauterine contraceptive device discontinuation among Indian women. J Obstet Gynaecol India. 2014;64(3):208–11. doi: 10.1007/s13224-014-0516-5 24966507 PMC4061341

[55] Hasanpoor-Azghdy SB, Simbar M, Vedadhir A. The Social Consequences of Infertility among Iranian Women: A Qualitative Study. Int J Fertil Steril. 2015;8(4):409–20. doi: 10.22074/ijfs.2015.4181 25780523 PMC4355928

[56] Asker C, Stokes-Lampard H, Beavan J, Wilson S. What is it about intrauterine devices that women find unacceptable? Factors that make women non-users: a qualitative study. J Fam Plann Reprod Health Care. 2006;32(2):89–94. doi: 10.1783/147118906776276170 16824298

[57] Okenyoru DS, Matoke V, Odhiambo F, Salima R, Anyika D, Ogutu G. Social-cultural factors influencing modern contraceptive uptake among women of the reproductive age in Turkana County, Kenya. Int J Community Med Public Health. 2023;11(1):51–6. doi: 10.18203/2394-6040.ijcmph20234107

[58] Gueye A, Speizer IS, Corroon M, Okigbo CC. Belief in Family Planning Myths at the Individual and Community Levels and Modern Contraceptive Use in Urban Africa. Int Perspect Sex Reprod Health. 2015;41(4):191–9. doi: 10.1363/4119115 26871727 PMC4858446

[59] Hidayat R, Callathea C, Jayadi TI, Wilson M. Knowledge, attitudes, and practices regarding contraception among young women in urban Indonesia: A mixed-methods approach. Sriwijaya J Obstet Gynecol. 2024;2(1):7–20. doi: 10.59345/sjog.v1i2.95

[60] Rahman MM, Islam AZ, Islam MR. Rural-urban differentials of knowledge and practice of contraception in Bangladesh. J Pop Soc Stud. 2010;18(2):87–110.

[61] Kamal SMM, Islam MA. Contraceptive use: socioeconomic correlates and method choices in rural Bangladesh. Asia Pac J Public Health. 2010;22(4):436–50. doi: 10.1177/1010539510370780 20659903

[62] Makins A, Taghinejadi N, Sethi M, Machiyama K, Munganyizi P, Odongo E, et al. FIGO postpartum intrauterine device initiative: Complication rates across six countries. Int J Gynaecol Obstet. 2018;143 Suppl 1:20–7. doi: 10.1002/ijgo.12600 30225873

[63] Mbizvo MT, Phillips SJ. Family planning: choices and challenges for developing countries. Best Pract Res Clin Obstet Gynaecol. 2014;28(6):931–43. doi: 10.1016/j.bpobgyn.2014.04.014 24957693

[64] Nalwadda G, Mirembe F, Tumwesigye NM, Byamugisha J, Faxelid E. Constraints and prospects for contraceptive service provision to young people in Uganda: providers’ perspectives. BMC Health Serv Res. 2011;11:220. doi: 10.1186/1472-6963-11-220 21923927 PMC3181204

[65] Rahman M, Haider MM, Curtis SL, Lance PM. The Mayer Hashi Large-Scale Program to Increase Use of Long-Acting Reversible Contraceptives and Permanent Methods in Bangladesh: Explaining the Disappointing Results. An Outcome and Process Evaluation. Glob Health Sci Pract. 2016;4 Suppl 2(Suppl 2):S122–39. doi: 10.9745/GHSP-D-15-00313 27540119 PMC4990156

[66] Khan MN, Harris M, Loxton D. Modern Contraceptive Use Following an Unplanned Birth in Bangladesh: An Analysis of National Survey Data. Int Perspect Sex Reprod Health. 2020;46:77–87. doi: 10.1363/46e8820 32401729

[67] Aziz MM, El-Gazzar AF. Provider bias and family planning in Upper Egypt: a simulated client approach. J Egypt Public Health Assoc. 2023;98(1):19. doi: 10.1186/s42506-023-00144-6 37777657 PMC10542042

[68] Sieverding M, Schatzkin E, Shen J, Liu J. Bias in Contraceptive Provision to Young Women Among Private Health Care Providers in South West Nigeria. Int Perspect Sex Reprod Health. 2018;44(1):19–29. doi: 10.1363/44e5418 30028307

[69] Chakraborty NM, Murphy C, Paudel M, Sharma S. Knowledge and perceptions of the intrauterine device among family planning providers in Nepal: a cross-sectional analysis by cadre and sector. BMC Health Serv Res. 2015;15:39. doi: 10.1186/s12913-015-0701-y 25627578 PMC4322443

[70] Hardee K, Jordan S. Advancing Rights-Based Family Planning from 2020 to 2030. Open Access J Contracept. 2021;12:157–71. doi: 10.2147/OAJC.S324678 34531690 PMC8438348

[71] Ontiri S, Ndirangu G, Kabue M, Biesma R, Stekelenburg J, Ouma C. Long-Acting Reversible Contraception Uptake and Associated Factors among Women of Reproductive Age in Rural Kenya. Int J Environ Res Public Health. 2019;16(9):1543. doi: 10.3390/ijerph16091543 31052372 PMC6539670

[72] Speizer IS, Hotchkiss DR, Magnani RJ, Hubbard B, Nelson K. Do service providers in Tanzania unnecessarily restrict clients’ access to contraceptive methods?. Int Fam Plann Perspect. 2000;26(1):13–42. doi: 10.2307/2648285

[73] van Nes F, Abma T, Jonsson H, Deeg D. Language differences in qualitative research: is meaning lost in translation? Eur J Ageing. 2010;7(4):313–6. doi: 10.1007/s10433-010-0168-y 21212820 PMC2995873

